# Supplementary material for: MetaRibo-Seq measures translation in microbiomes
Source: Nat Commun. 2020 Jun 29;11:3268. doi: 10.1038/s41467-020-17081-z (PMC7324362; doi:10.1038/s41467-020-17081-z)
Supplement: Supplementary file 10 — Supplementary Data 7 [file 41467_2020_17081_MOESM10_ESM.zip › File2/Confidence_High_Taxonomy/95503_out.krona.html]

Javascript must be enabled to view this page.

members
magnitude
magnitudeUnassigned
count
unassigned
taxon
rank

95503\_out

2

2
2
superkingdom

1
976
phylum

1
200643
class

171549
order
1

family
1853231
1

283168
genus
1

species
28118
1

SRS144714\_contig\_number\_contig-100\_8392.122331

1239
phylum
1

1
class
91061

order
186826
1

1
1300
family

genus
1301
1

1

SRS057619\_contig\_number\_11885
1305
species
